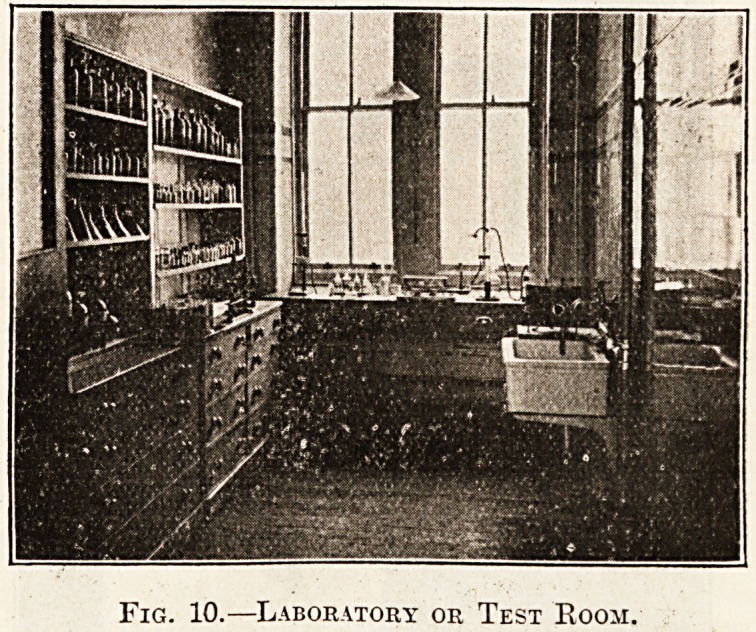# The Units of General Hospital Construction

**Published:** 1907-05-18

**Authors:** 


### HOSPITAL ADMINISTRATION.

### CONSTRUCTION AND ECONOMICS.

### THE UNITS OF GENERAL HOSPITAL CONSTRUCTION.

II. THE WARD UNIT (*concluded*).

The next illustration (fig. 6) is that of the ward kitchen, 13 feet by 9 feet, and its fittings are worthy

Fig. 6.—Ward Kitchen.

of careful study. The window is of practically the same dimensions as those of the ward. The bossing

Fig. 7.—Cleaners' Scullery.

is utilised as a cupboard, and the cupboard has a ventilating opening guarded externally by louvres, and internally by an iron frame covered over with gauze. As shown in the illustration, the doors of the cupboard being open, the milk, butter, etc., for ward use are stored here, where they are free from dust and the heat of the kitchen. In the right-hand corner, next the window, is the sink with a teak drip-board attached. The sink is supplied with hot and cold water through a junction tap, and check valves are placed on both hot and cold water pipes, so that repairs may be carried out on the taps without emptying the pipes in other portions of the building. On the same side is a small coal range.

Fig. 8.—Linen Room.

On the opposite wall is a dresser on wheels, which facilitates its being moved from the wall for the purpose of cleaning. Above the dresser are three cupboards with sloping tops, fixed to the wall by means of galvanised iron brackets. One of these cupboards is of iron and used for the storage of bread, the doors and sides being perforated for ventilation. The second is fitted with racks and pegs for crockery. The third is for the private use of the nurse.

It may be noted that there is no inspection window into the ward. This is not considered necessary, nor is it desirable in a ward with acute cases, as only a limited number of beds can be within view. In a well administered hospital the inspection window has become obsolete, and it has the structural disadvantage of breaking the wall-surface and adding ledges where dust may collect.

The next illustration (fig. 7) is that of the cleaners' scullery, 12 feet by 5 feet, adjoining the ward kitchen, and containing a coal-box on wheels, a sink, similar to that in the ward kitchen, with hot and cold water tap placed high enough to let a bucket be filled with ease, a rack for brushes and a basket for dusters, etc. Everything is exposed, and there are no corners for articles to be hidden or dirt to collect.

Fig. 8 illustrates the room for the storage of the ward linen, 12 ft. by 6 ft. 6 in. This room is fitted up with presses having sparred shelves, and ventilated top and bottom. The doors are made to slide on metal rollers in metal grooves. It is an advantage to have all the main heating-pipes passing through this room, as it ensures everything being kept thoroughly dry.

Fig. 9.—Patent Swivel Bath.

The patients' lavatory is 13 ft. by 5 ft. 10 in., with bathroom adjoining, and built out from it is a private lavatory for nurses, 4 ft. by 3 ft. 6 in. The patients' lavatory contains two basins with hot and cold water supply. On the opposite wall is a portable bath with an arrangement for filling and emptying. This bath is on four wheels, and can be removed to the bedside when necessary. Over the portable bath is placed a metal rail 5 ft. from the floor, and projecting 1 ft. from the wall, where mackintosh sheeting and jaconet may be hung up to dry. Adjoining the lavatory is the patients' bathroom. The bathroom, 9 ft. 6 in. by 6 ft. 10 in., is placed at the entrance to the ward, so that a patient may be bathed before admission without requiring to pass through the ward. The objection which is sometimes raised to having the bathroom placed in this position is that it occupies too much space, and requires additional drain pipes. By adopting the bath here illustrated, which has been specially designed for this ward unit, a large bathroom is not required. It is necessary that the nurse or attendant should be able to get on either side of a hospital bath, and by means of the swivel arrangement this can be accomplished in comparatively small space.

The bath is supplied with hot and cold water through a special valve fixed on the wall, worked by a movable key. The key can be removed by the nurse, so that patients cannot waste water by leaving it turned on. The valve is arranged so that cold, hot, or tepid water can be had as required, but the cold supply must be turned on first, in this way preventing any possibility of scalding the patient The drains for the bathroom and lavatory are disconnected from all sewage pipes, and are exposed on the outside wall.

The ward sisters' duty room is 12 ft. by 8 ft. 3 in. Here she keeps her books and interviews her nurses. The furnishings are simple, consisting of a writing table with row of drawers, a corner cupboard, an easy chair, and two small chairs.

The visiting physician's private room is used for interviews with his assistants, and here he keeps the charts and records of his cases and any instruments, such as microscopes, required for teaching purposes.

Fig. 10 illustrates the laboratory or test-room. This room should have a large window without any mullion, so that there is no obstruction to light. It is fitted with a sink with hot and cold water, and a centrifuge worked by water pressure from the cold water pipe. On the opposite wall are cupboards with shelves for test and stock bottles, and drawers of various sizes for the storage of diagrams, charts, etc.

In order that convalescents may be more easily supervised, and that no light or air may be cut off from the corridor, the day room is simply divided from the corridor by a plain wood partition 4 ft. 6 in. high at the centre and 7 ft. at the end. Here the convalescents have their food, and they can amuse themselves without disturbing the patients in the ward.

The washing and examination-room is used for the examination of patients who have been in the hospital and return to be seen by the visiting physician. It is also used for the thorough washing of patients before being taken into the ward who cannot be put in a bath. A porcelain slab moving on a pivot, similar to the arrangement for the revolving bath, is fixed in one corner of the room, and a swivel bath in the other. The room is specially heated, so that there is no danger of the patients catching cold. The provision of this room does away with the objectionable practice of taking return patients into the ward and placing them in beds for purposes of examination without being properly bathed; it also allows the nurse to get the patient thoroughly cleaned before admission. There is no doubt that vermin, if not infection, is sometimes introduced into a ward when return cases are admitted for examination, and the provision of such a room as is here described does away with any such possibility.

Fig. 10.—Laboratory or Test Room.

## Figures and Tables

**Fig. 6. f1:**
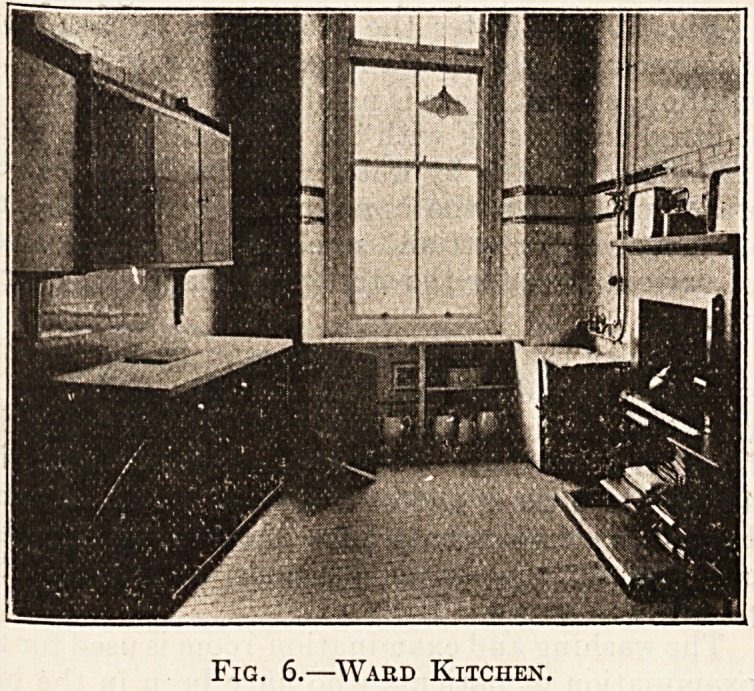


**Fig. 7. f2:**
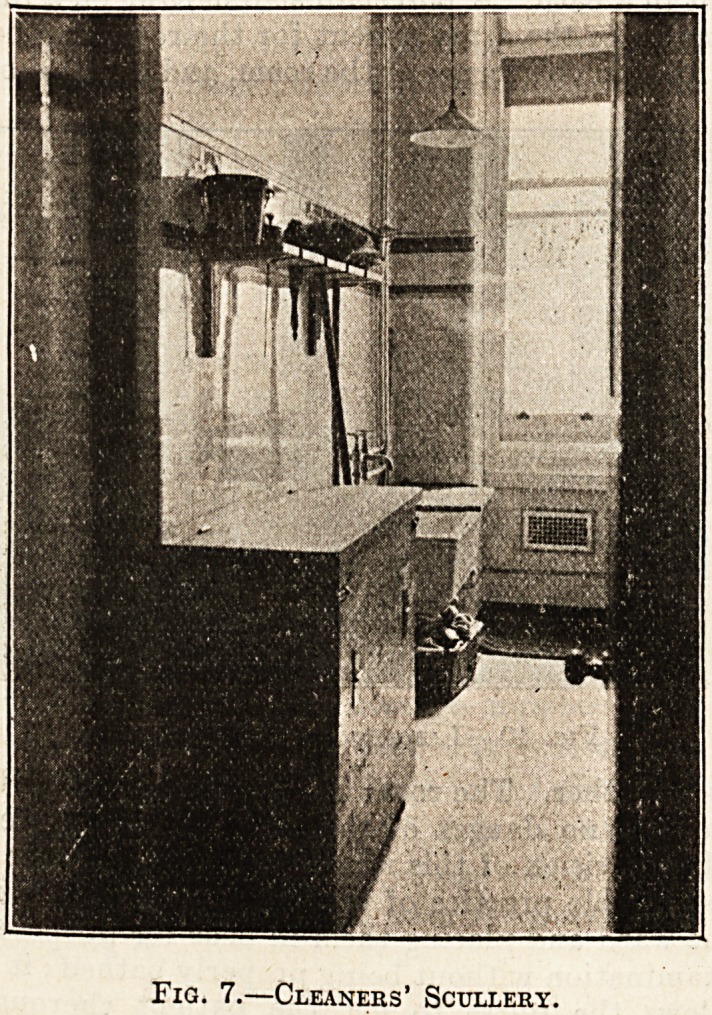


**Fig. 8. f3:**
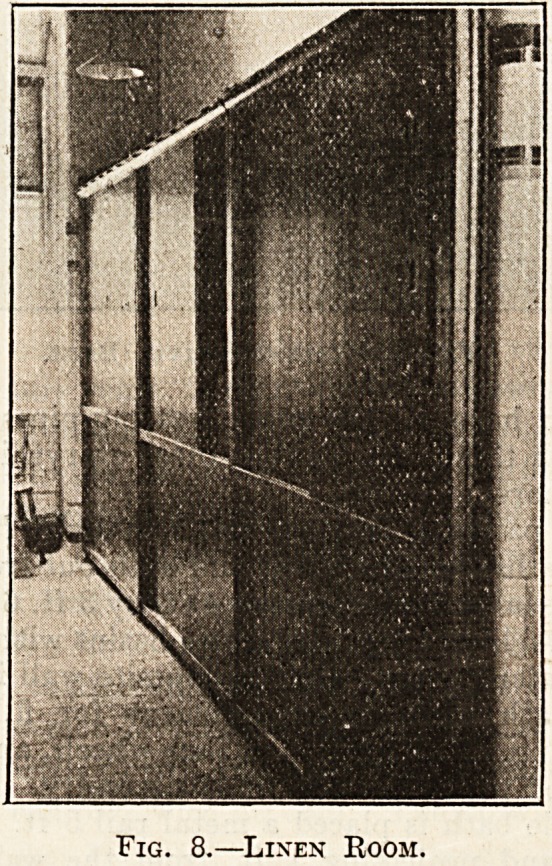


**Fig. 9. f4:**
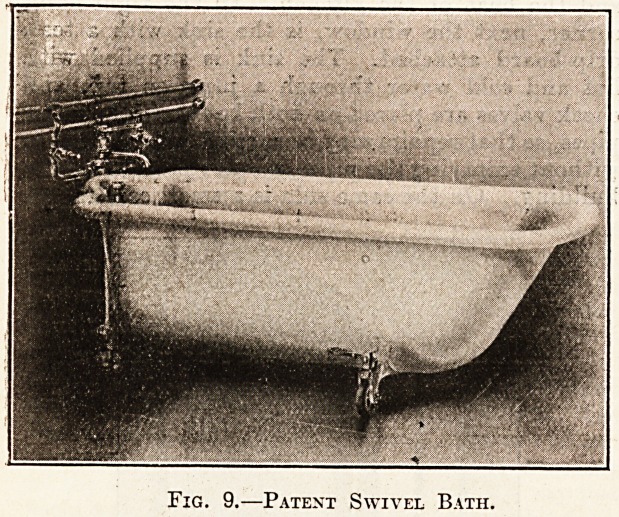


**Fig. 10. f5:**